# Identification of Unknown Biological Toxin Proteins Using Mass Spectrometry: A Case Study on De Novo Sequencing of Ricin

**DOI:** 10.3390/toxins17110564

**Published:** 2025-11-18

**Authors:** Yubo Song, Hao Wang, Junjie Wen, Jiale Xu, Siyu Zhu, Fuli Wang, Yongqian Zhang

**Affiliations:** 1 State Key Laboratory of Chemistry for NBC Hazards Protection, Beijing 102205, China; 2 School of Medical Technology, Beijing Institute of Technology, Beijing 100081, China

**Keywords:** ricin, heuristic de novo sequencing, mass spectrometry, biological toxin protein identification

## Abstract

Background: The rapid and reliable identification of unknown or highly variable biological toxin proteins, such as the potent Ricin toxin, remains a critical challenge in biodefense and public security. Methods: To address this, we developed a Heuristic De Novo Sequencing (HDPS) strategy, which combines multiple enzymatic and microwave-assisted acid hydrolysis to generate diverse peptides, followed by a two-stage assembly process integrating de novo sequencing with homology-based database searching for robust error correction. Results: When applied to Ricin, this approach achieved 100% sequence coverage for both its A and B chains, with amino acid-level accuracies of 98.13% and 98.47%, respectively, and successfully corrected potential sequencing ambiguities. Conclusions: These results demonstrate that HDPS is a highly accurate and effective method for the de novo sequencing of full-length proteins, making it particularly valuable for characterizing unknown or mutated toxins in the absence of comprehensive reference databases.

## 1. Introduction

Ricin is a heterodimeric, type II ribosome-inactivating protein derived from *Ricinus communis*. Its A chain catalytically depurinates rRNA, thereby irreversibly inhibiting protein synthesis, whereas the B chain facilitates cellular entry through glycan-binding and endocytosis [1,2,3]. Following internalization, the toxin undergoes retrograde transport to the endoplasmic reticulum (ER), where the chains are separated, releasing the cytotoxic A chain into the cytosol [4,5]. Due to its high toxicity and relative accessibility, ricin is considered a potential bioterror agent [6]. Notably, its components also hold therapeutic promise: the A chain can be used in immunotoxins, and the B chain may serve as a targeting carrier for nanoparticles, illustrating a dual-use potential [7]. Consequently, sequencing and biologically analyzing such toxins are vital for advancing both countermeasures and biomedical applications [8,9].

Currently, detection technologies for protein-based biological toxins are broadly divided into two categories. The first category includes immunoassays that employ specific antibodies, such as enzyme-linked immunosorbent assay (ELISA), Western blot (WB), immunochromatographic assay (ICA), colloidal gold immunochromatographic strip (CGIS), and electrochemiluminescence-based methods [10,11,12,13]. Although these methods offer high sensitivity and operational simplicity, their performance heavily relies on the specificity and affinity of the antibodies used. The second category encompasses mass spectrometry (MS)-based techniques, which accurately determine amino acid sequences of toxin-specific peptides by interpreting MS data. For example, Sousa et al. first reported the use of accelerated solvent extraction (ASE) to process complex samples containing Ricin. Subsequent tryptic digestion and MS analysis led to the identification of 19 peptides, with the tandem mass spectrometry (MS/MS) spectra of three peptides confirming the uniqueness of Ricin [14]. Separately, Chen et al. developed a microwave-assisted acid hydrolysis (MAAH) method, which utilizes hot acid to selectively cleave aspartic acid residues, reducing hydrolysis time to just 15 min and significantly improving efficiency; subsequent MS analysis also successfully identified disulfide-bonded peptides within the Ricin sequence [15]. Despite the diversity of available detection technologies, a universally applicable standard reference method is still lacking [16].

MS-based de novo sequencing adopts a bottom-up strategy to determine the full-length sequences of unknown biological toxin proteins. The process begins with enzymatic digestion of the target protein into peptides, followed by MS/MS analysis of their fragmentation patterns. The resulting spectral data are then assembled de novo, typically utilizing a de Bruijn graph algorithm, which constructs contigs from overlapping peptide segments. This theoretical framework facilitates the accurate reconstruction of the primary protein structure. However, in practice, instrumental signal loss and background noise in MS data can mislead de novo algorithms, resulting in erroneous sequence assignments [17]. Furthermore, the de Bruijn graph assembly is highly dependent on the depth and accuracy of the peptide sequencing; low coverage often leads to fragmented assembly and prematurely truncated contigs [18]. The conventional single-step de novo assembly strategy is highly prone to the transmission and accumulation of such errors, ultimately leading to a reduction in the accuracy of the protein sequence.

To address these limitations, we developed a Heuristic De Novo Protein Sequencing (HDPS) technology. This technology combines microwave-assisted and enzymatic hydrolysis to significantly improve the diversity and yield of peptide sequences. After initial construction of sequence overlap scaffolds, HDPS implements a two-stage assembly and error-correction strategy that integrates graph theory with database searching, thereby achieving highly robust protein sequence assembly [19,20]. By employing Ricin as a model system, we validated the high accuracy and reliability of the HDPS platform for full-length toxin protein sequencing. Our method is designed to overcome persistent challenges in traditional MS-based identification, notably extended cycle times and assembly difficulties, thereby achieving over 95% accuracy at the amino acid level. It thus provides a powerful and versatile tool for characterizing protein toxins from novel or unknown sources [21,22,23,24].

## 2. Results and Discussion

**Figure 1:**
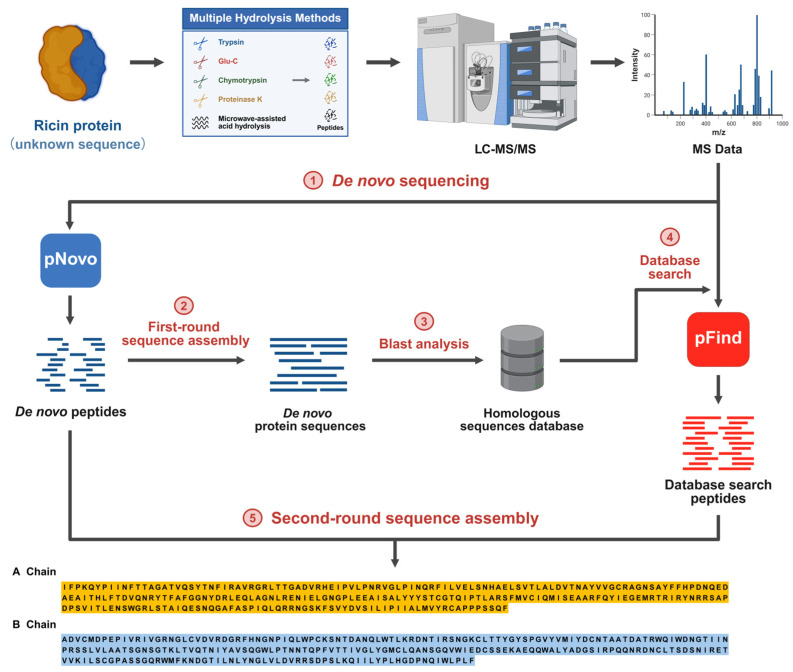
Workflow of the Heuristic De Novo Protein Sequencing (HDPS) technique.

MS-based peptide de novo sequencing and sequence assembly are recognized as pivotal technologies for determining the full-length sequence of proteins at the amino acid level. It plays a crucial role in various fields, including identifying amino acid mutations, discovering biomarkers for disease treatment response, and screening novel therapeutic agents [24,25,26]. In this study, we integrated mass spectrometry-based de novo protein sequencing with database searching, naming the combined approach HDPS. The technical workflow is detailed in Figure 1. The process began with the digestion of the Ricin protein (as an unknown sequence) using multiple enzymatic digestions combined with microwave-assisted acid hydrolysis to ensure comprehensive sequence coverage. The resulting peptides were analyzed by MS/MS to acquire fragmentation spectra. Subsequently, de novo sequencing was performed on the MS/MS data using pNovo software, and an initial assembly round yielded preliminary sequence contigs. These contigs were then subjected to homology analysis to construct a custom sequence database, which was searched against using pFind software to identify high-confidence peptide-spectrum matches. Finally, peptides from both the de novo and database search results were consolidated and subjected to a second, integrated assembly round, ultimately determining the complete amino acid sequence of the Ricin protein.

### 2.1. Peptide De Novo Sequencing

**Figure 2:**
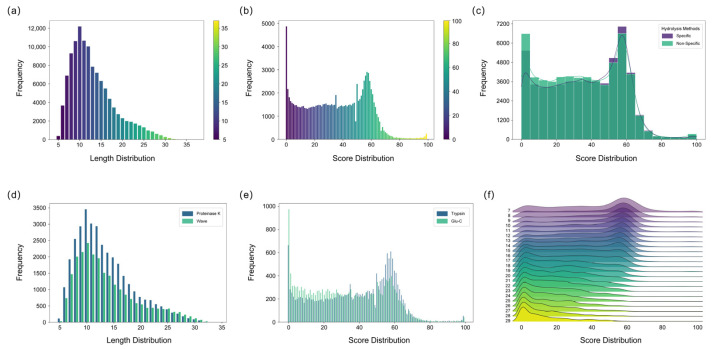
Characterization of de novo sequencing results for Ricin toxin peptides using five hydrolysis methods. (**a**) Length distribution results of de novo sequencing peptides analyzed by five hydrolysis methods. (**b**) Score distribution results of de novo sequencing peptides analyzed by five hydrolysis methods. (**c**) Score distribution comparison between specific and nonspecific hydrolysis methods. (**d**) Length comparison of peptides from the Proteinase K and MAAH methods (**e**) Score comparison of peptides hydrolyzed by Trypsin and Glu-C. (**f**) Combined score and length distribution of de novo sequencing peptides of Ricin toxin.

The amino acid sequences of peptides were directly determined from the mass spectrometry data using de novo sequencing algorithms. The peptide lengths identified by the pNovo software were predominantly distributed between 6 and 18 amino acids, representing an optimal length range suitable for the subsequent sequence assembly process (Figure 2a) [27,28]. However, the accuracy of de novo sequencing is inherently constrained by factors such as instrumental noise and incomplete peptide fragmentation, often leading to errors including the misidentification of isobaric amino acids [29]. Therefore, when sequencing unknown proteins, rigorous quality control of the de novo sequencing results is essential to reduce the complexity of subsequent peptide assembly. Overall, the majority of the de novo sequenced peptides were associated with confidence scores below 60, underscoring the limited per-segment accuracy of the method and necessitating validation and correction via subsequent database searching to ensure the assembly of correct sequences (Figure 2b). Consequently, a threshold based on the de novo sequencing score was applied to filter and remove low-confidence peptide assignments initially, thereby enhancing the accuracy and robustness of the final sequence assembly.

This study employed both specific and non-specific hydrolysis methods. Generally, the non-specific method yielded a greater quantity of peptides and a higher proportion of usable peptides, indicating its efficacy in enhancing peptide diversity and overlap, which is crucial for sequence assembly (Figure 2c). In contrast, specific enzymatic digestion cleaves at predictable sites and requires a relatively stable, controllable digestion time. Non-specific digestion, lacking fixed cleavage sites, necessitates strict control of digestion duration to prevent over-digestion, which could generate excessively short peptides detrimental to assembly [30]. Both Proteinase K digestion and microwave-assisted acid hydrolysis exhibited high non-specificity, generating peptides with substantial sequence overlap. Proteinase K yielded a higher number of peptides than MAAH, likely attributable to the challenges in precisely controlling the temperature during MAAH compared to the more stable enzymatic conditions (Figure 2d). Although peptides derived from specific enzymatic digestion generally exhibit higher per-residue accuracy and slightly elevated confidence scores in de novo sequencing, they are considerably fewer in number compared to those generated by non-specific digestion. Moreover, the sequence overlap achievable with specific-digestion peptides is inherently limited by the distribution of the enzyme’s cleavage sites along the protein sequence. Among specific proteases, trypsin outperformed Glu-C, yielding a greater number of peptides with higher overall scores (Figure 2e). This advantage can be attributed to the superior fragmentation efficiency of tryptic peptides during mass spectrometry, which produces more continuous b- and y-ion series, thereby significantly enhancing the accuracy of de novo sequencing [29,31].

To assess how peptide characteristics influence the sequencing results, the correlation between peptide length and confidence scores was examined. The de novo sequenced peptides for Ricin toxin exhibited a length distribution of 7–29 amino acids and confidence scores ranging from 0 to 100. A clear inverse correlation was observed between peptide length and confidence score, wherein longer peptides generally corresponded to lower scores (Figure 2f). This trend can be attributed to the higher probability of fragment ion loss in longer peptides, which compromises sequencing accuracy. In contrast, shorter peptides typically provide more complete b-/y-ion coverage and exhibit better spectral continuity, resulting in more reliable sequence inference. While longer peptides theoretically reduce the number of assembly steps, they become less favorable when sequencing accuracy is limited. Conversely, extremely short peptides increase the risk of random k-mer repetition and are also suboptimal for assembly. To balance these factors, a minimum k-value of 6 was adopted in this study to enhance the robustness and efficiency of the subsequent sequence assembly process.

### 2.2. First-Round Sequence Assembly

**Figure 3:**
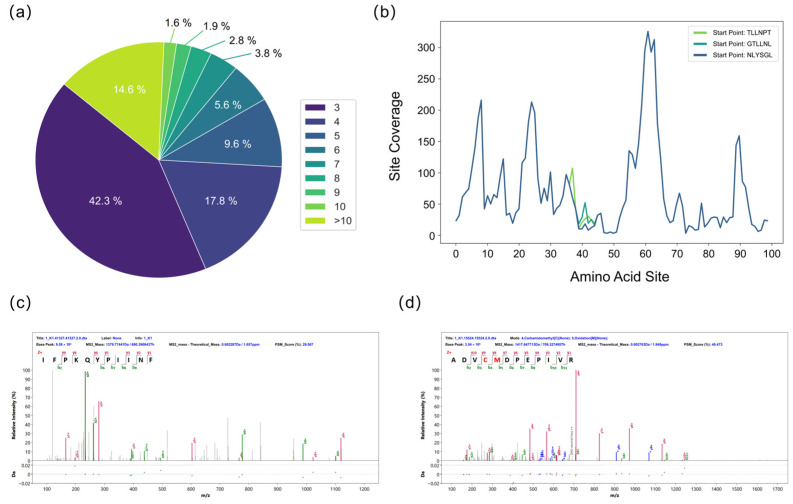
Evaluation during the splicing process. (**a**) Frequency distribution of short k-mers from de novo sequencing used to assess k-mer credibility. (**b**) The top three assembled sequences and site-specific confidence scores for the Ricin B chain after the first assembly round. (**c**) MS/MS spectrum of the N-terminal sequence of the A chain (IFPKQYPIINF). (**d**) MS/MS spectrum of the N-terminal sequence of the B chain (ADVCMDPEPIVR).

To mitigate errors inherent in de novo sequencing, further filtering of the peptide sequences was necessary. Each de novo sequenced peptide was segmented into K-mers, the credibility of which was evaluated based on their frequency of occurrence. A high-frequency K-mer typically indicates that its sequence is supported by abundant, mutually verifying fragment ion information across multiple MS/MS spectra, signifying high confidence. In contrast, K-mers derived from erroneous peptide sequences exhibit low frequencies. Filtering out K-mers below a specified frequency threshold effectively removes these error-prone sequences (Figure 3a). The assembled sequences from different starting points were aligned. A majority voting principle was then applied at each amino acid position to identify and correct errors, such as isomeric amino acid pairs (e.g., GG=N and GA=Q), amino acid inversions (e.g., ES=SE), deamidation (e.g., Q/E, N/D), and isomeric forms of I and L [32]. The frequency score of each amino acid residue was calculated to assess the local confidence of the full-length sequence. Multiple initiation points were used for assembly to reconstruct the complete protein sequence. The sequences generated from these different starting points showed high overall similarity but contained variations at specific local sites (Figure 3b). Homology analysis of the initially assembled sequences confirmed that the protein consisted of the A and B chains of Ricin toxin. Because the de novo-derived peptides were randomly mixed and lacked positional information, we conducted homology analysis of the first-round sequence assembly to identify the N-terminal regions of the Ricin A and B chains, thereby establishing reliable initiation sites for sequence reconstruction. Figure 3c,d display representative MS/MS spectra of the N-terminal sequences of the A chain (IFPKQYPIINF) and the B chain (ADVCMDPEPIVR). Despite the error correction procedures, the initially assembled A and B chain sequences remained incomplete. This was attributed to factors such as insufficient peptide overlap at specific amino acid sites, unexpected modifications, glycosylation sites, and enzyme cleavage biases, all of which can compromise the accuracy of de novo peptide sequencing.

### 2.3. Second-Round Sequence Assembly

**Figure 4:**
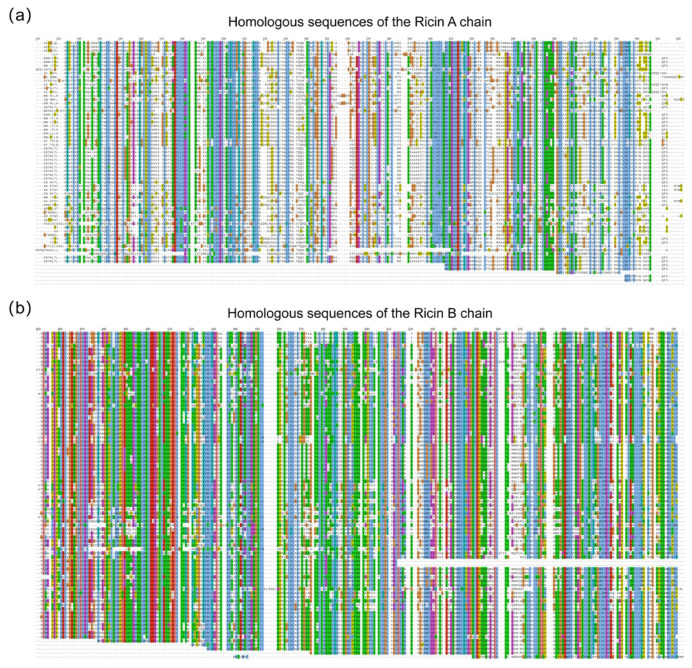
Comparative analysis of homologous sequences for the Ricin A and B chains. (**a**) Alignment of homologous sequences for the Ricin A chain (amino acids 110–420). (**b**) Alignment of homologous sequences for the Ricin B chain (amino acids 450–745).

To enhance the accuracy of sequence assembly, homologous sequences of the Ricin toxin A and B chains were identified by BLAST analysis, and peptides obtained through homologous sequence database searching were utilized in the second-round assembly. A total of 193 homologous sequences were successfully matched. Figure 4a,b illustrate amino acids 110–420 of the A chain and 450–745 of the B chain, respectively, encompassing regions contributing to the final protein sequencing results. At the level of individual amino acid residues, the number of matched homologous sequences serves as an indicator of evolutionary conservation and structural stability. Alignment analysis revealed that the B chain exhibits a high degree of conservation, whereas the A chain displays pronounced variability among homologs. This alignment information was further employed to resolve ambiguous isobaric amino acid residues—such as leucine and isoleucine—that cannot be distinguished by de novo sequencing alone. It should be noted that residues in low-coverage regions of the homologous alignment may be unreliable owing to possible sequence mutations.

**Figure 5:**
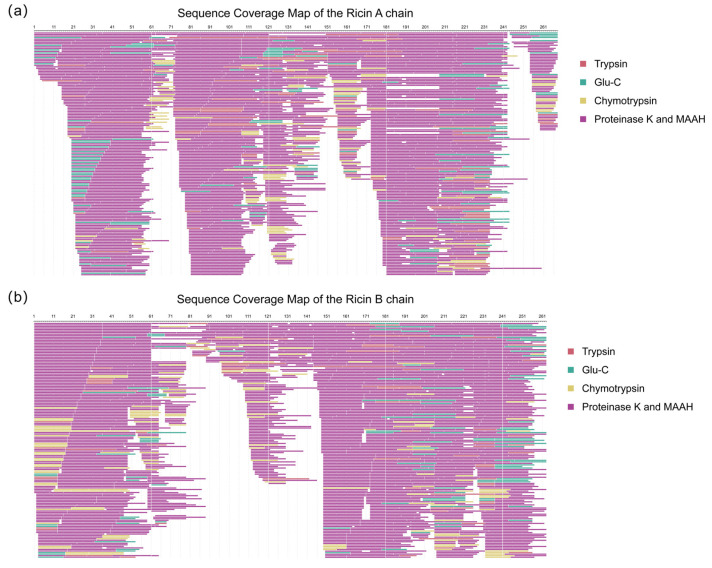
Sequence coverage map of the second-round assembly. (**a**) Ricin toxin A chain (267 amino acids), achieving complete (100%) sequence coverage. (**b**) Ricin toxin B chain (262 amino acids), achieving complete (100%) sequence coverage. The different colors of the covered peptides indicate the types of enzymatic digestions.

Subsequently, peptide sequences from both de novo sequencing and the database search were merged for a second sequence assembly. The assembled sequences were then aligned with homologous references, yielding the complete Ricin A and B chains. The assembly quality was assessed by calculating sequence coverage, which was confirmed to be 100% for both chains, as presented in Figure 5. The contributions of different digestion types to sequence coverage were not uniform, with non-specific cleavages (including proteinase K and MAAH) providing a greater contribution to overall coverage. The peptide sequences used for the second-round assembly are listed in Table S5.

**Figure 6:**
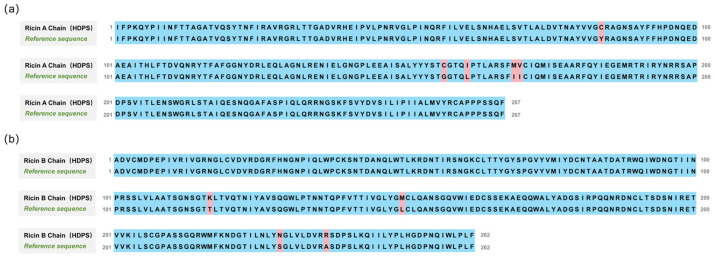
Alignment of the amino acid sequences of Ricin toxin obtained via HDPS with the reference sequence (UniProt P02879, NCBI GI:132567). (**a**) Ricin A chain alignment, achieving 98.13% sequence accuracy. (**b**) Ricin B chain alignment, achieving 98.47% sequence accuracy.

Figure 6 presents the alignment of the amino acid sequences of Ricin toxin obtained via the HDPS method against the corresponding reference sequence (UniProt P02879, NCBI GI:132567) [33]. The HDPS approach achieved full-length coverage for both chains, exhibiting an accuracy of 98.13% (262/267) for the A chain and 98.47% (258/262) for the B chain. Moreover, the method correctly distinguished isobaric amino acid residues (I/L) with an accuracy of 95.40% (83/87), further demonstrating its exceptional reliability and high precision in reconstructing complex protein sequences.

HDPS is a novel sequence assembly strategy that uniquely integrates homology-based database searches with a two-round sequence assembly, thereby enhancing the utilization of informative peptides derived from mass spectrometry data. A comparative evaluation was performed between the HDPS framework and the established ALPS software [24,34]. As shown in Table 1, both platforms achieved complete sequence coverage for the Ricin A and B chains. Nevertheless, HDPS exhibited higher sequencing accuracy, reaching 98.13% for the A chain and 98.47% for the B chain, compared with 95.88% and 95.80% obtained by ALPS. This systematic comparison demonstrates that HDPS enhances full-length protein sequencing coverage and accuracy while providing a robust approach for characterizing proteins with limited or unknown reference information.

## 3. Conclusions

In this study, a combination of specific and nonspecific enzymatic cleavage methods was used to increase the diversity of peptides derived from an unknown toxin protein, thereby facilitating sequence assembly by providing sufficient overlap. Starting from de novo sequencing of the peptide segments, secondary splicing was performed by integrating homology search and database identification, resulting in the full-length restoration of the complete amino acid sequence of the toxin. By incorporating peptide coverage information obtained at different PSM confidence levels from enzymatic cleavage, the reliability of the assembled sequence was evaluated in an intuitive manner, offering a novel strategy for identifying unknown biological toxins. The accuracy of de novo peptide sequencing remains challenging and has room for further improvement. Combining homologous sequences to identify novel proteins with extensive mutations constitutes a useful strategy, as exemplified in antibody sequencing studies. Additionally, unexpected post-translational modifications, such as glycosylation, can significantly affect the accurate assignment of peptide sequences during de novo sequencing. Although we have obtained supporting peptide evidence through homologous sequence searches, further optimization is needed to enhance the accuracy of de novo sequencing, particularly in the presence of unanticipated modifications, including glycosylation. Overall, we have established a comprehensive and flexible full-length protein sequencing strategy capable of accurately characterizing multi-subunit toxins with unknown sequences, which is particularly valuable for identifying engineered or naturally occurring toxin variants as well as novel toxins not yet represented in existing databases. Moreover, the HDPS strategy demonstrates broad applicability in protein identification and full-length protein sequencing, providing a reliable and scalable approach for addressing complex challenges in protein characterization.

## 4. Materials and Methods

### 4.1. Sample Preparation

The sterile Ricin protein was purchased from Beijing Hapten and Protein Biomedical Institute, Beijing, China. It was purified by affinity chromatography at a concentration of 2 mg/mL and subsequently processed via enzymatic digestion and MAAH. For enzymatic digestion, Ricin was digested using four proteases: Trypsin (Promega, V511A), Glu-C (Promega, V1651), Chymotrypsin (Promega, V1061), and Proteinase K (Promega, V3021). The sample was first loaded into a 10 kDa molecular weight cut-off ultrafiltration centrifuge tube (Sartorius, VN01H02) [35]. After centrifugation, denaturation was performed using 8 M urea, followed by reduction with dithiothreitol (DTT) and alkylation with chloroacetamide (CAA). The final concentrations of DTT and CAA were 10 mM and 50 mM, respectively. Enzymatic digestion was carried out under the following conditions: Trypsin and Glu-C digestions were performed in 50 mM ammonium bicarbonate (pH 7.8) at 37 °C for 18 h with an enzyme-to-substrate ratio of 1:50 (*w*/*w*). Chymotrypsin digestion was performed in 100 mM Tris-HCl (pH 8.0) at 25 °C for 18 h with an enzyme-to-substrate ratio of 1:50 (*w*/*w*). Proteinase K digestion was performed in 50 mM Tris-HCl (pH 8.0) at 37 °C for 5 min and 20 min at an enzyme concentration of 50 µg/mL [36]. All digestion reactions were quenched by adding formic acid. After digestion, the peptide solution in the ultrafiltration tube was collected by centrifugation and vacuum-dried at 60 °C. Prior to MAAH, Ricin was similarly subjected to denaturation, reduction, and alkylation. The buffer was then exchanged for pure water, and the sample was transferred to a glass vial. Hydrochloric acid was added to a final concentration of 3 M, and microwave irradiation was applied at 700 W for 4 min with ice insulation, with the ice replenished every minute. The hydrolyzed sample was then desalted and vacuum-dried.

### 4.2. Liquid Chromatography and Mass Spectrometry Analysis

The digested peptides were dissolved in 0.1% formic acid and analyzed online using an Easy-nLC 1200 HPLC system (Thermo Fisher Scientific, Waltham, MA, USA) coupled to an Orbitrap Q-Exactive HF mass spectrometer (Thermo Fisher Scientific), with separation on a self-packed C18 column (15 cm × 150 μm, 1.9 μm particle size) via gradient elution. A 60 min elution gradient was applied. Mobile phase A consisted of 0.1% formic acid in water, and mobile phase B consisted of 80% acetonitrile and 0.1% formic acid in water. The gradient program was as follows: 4% B at 0 min, 7% B at 1 min, 13% B at 5 min, 25% B at 35 min, 45% B at 53 min, 95% B at 54 min, and held at 95% B until 60 min. The flow rate was set to 600 nL/min. Mass spectrometry data were acquired in data-dependent acquisition (DDA) mode. Full-scan MS spectra (350–2000 *m*/*z*) were acquired at a resolution of 60,000, with an AGC target of 3 × 10^6^ and a maximum injection time of 30 ms. The top 20 most intense precursor ions were selected for fragmentation. MS/MS spectra were acquired at a resolution of 15,000, with an isolation window of 1.6 *m*/*z*, an AGC target of 5 × 10^4^, a maximum injection time of 45 ms, a normalized collision energy of 27%, a dynamic exclusion of 45.0 s, a charge exclusion of 1, 8, >8, and an intensity threshold of 1.8 × 10^4^.

### 4.3. Peptide De Novo Sequencing

Raw data files conversion was performed with ProteoWizard’s msConvert (version 3.0.24026), with a binary encoding precision of 64 bits. For the mgf conversion, centroiding was performed using the vendor’s peak picking algorithm included in msConvert. De novo sequencing was performed using pNovo software (version 3.1.5). For data from Trypsin, Glu-C, and Chymotrypsin digestions, the corresponding specific enzymatic cleavage sites were selected. For data from Proteinase K digestion and MAAH, non-specific cleavage was selected. Higher-energy collisional dissociation (HCD) was selected as the fragmentation mode. The precursor mass tolerance was set to ±10 ppm, and the fragment ion mass tolerance was set to ±0.02 Da. Carbamidomethylation [C] was set as a fixed modification, while Oxidation [M], Deamidation [N] and Deamidation [Q] were set as variable modifications. De novo sequencing results for peptides were obtained by searching the MGF files corresponding to the five hydrolysis methods.

### 4.4. First-Round Sequence Assembly

Peptides with a PSM score greater than 20 from the de novo sequencing results were retained. Each peptide was segmented into shorter fragments of a fixed length K (K-mers) using a sliding window. The occurrence frequency (R) of each K-mer was calculated, and only K-mers with a frequency greater than 2 were retained for assembly. De novo sequencing results from multiple enzymatic digestions were merged. The top 3000 most frequent K-mers were selected as starting points for assembly. Extension was performed by searching for overlapping K-mers (K-1 overlap) from both ends. Only assembled sequences with lengths between 50 and 400 amino acids were retained.

### 4.5. Homologous Sequences Database Search

The first assembled sequences were subjected to homology analysis using the Protein BLAST tool on the NCBI website (https://blast.ncbi.nlm.nih.gov/ accessed on 10 October 2024). The obtained protein sequences were compiled into a custom homologous sequence database. Database searching against the custom database was performed on the raw files from the multiple digestions using pFind software (version 3.2.0). Search parameters were consistent with those used for pNovo.

### 4.6. Second-Round Sequence Assembly

Peptide sequences identified from the database search offer higher accuracy but cannot detect sequence mutations or unknown proteins. Therefore, peptides from both de novo sequencing and database search results were combined for a second round of sequence assembly. Peptides with a PSM score > 20 from de novo results and peptides with an FDR < 1% from database search results were retained. The K-mer length was set to 7. The top 100 most frequent K-mers were used as assembly initiation points. K-mers with a frequency greater than 2 were used for assembly, and unique sequences with lengths between 50 and 400 amino acids were retained. Assembled sequences from different starting points showed high similarity, differing only at specific local sites. A sequence alignment method was employed to align the amino acids at each position. The frequency of occurrence of each amino acid at a given position was used as an assembly confidence score for that site. This information was utilized to correct errors such as isobaric amino acid assignments and amino acid rearrangements.

## Figures and Tables

**Table 1:** Assembly performance comparison between ALPS and HDPS using de novo sequenced peptides.

	Ricin A Chain		Ricin B Chain	
	ALPS	HDPS	ALPS	HDPS
Coverage	100%	100%	100%	100%
Accuracy	95.88%	98.13%	95.80%	98.47%

## Data Availability

All the mass spectrometry proteomics data associated with this study have been deposited at the ProteomeXChange Consortium via the PRIDE repository with identifier PXD061213. You can access the dataset by logging in to the PRIDE website using the following account details. Username: reviewer_pxd061213@ebi.ac.uk, Password: UDBL8vKulBkt.

## Supplementary Materials

The following supporting information can be downloaded at: https://www.mdpi.com/article/10.3390/toxins17110564/s1, Table S1: Peptide De Novo Sequencing Results; Table S2: Score Distribution of De Novo Sequencing Results; Table S3: Length Distribution of De Novo Sequencing Results; Table S4: Database Search Peptides; Table S5: Peptide Sequences Used for the Second-Round Assembly; Table S6: Chromatography and Mass Spectrometry Parameters.
